# Folic acid-conjugated dextran-coated Zn_0.6_Mn_0.4_Fe_2_O_4_ nanoparticles as systemically delivered nano heaters with self-regulating temperature for magnetic hyperthermia therapy of liver tumors

**DOI:** 10.1038/s41598-023-40627-2

**Published:** 2023-08-21

**Authors:** Meysam Soleymani, Amirhoushang Poorkhani, Solmaz Khalighfard, Mohammad Velashjerdi, Vahid Khori, Saeed Khodayari, Hamid Khodayari, Mohammad Dehghan, Nazila Alborzi, Shahram Agah, Ali Mohammad Alizadeh

**Affiliations:** 1 Department of Chemical Engineering, Faculty of Engineering, Arak University, Arak, 38156-88349 Iran; 2 Ischemic Disorders Research Center, Golestan University of Medical Sciences, Gorgan, Iran; 3 Research Center on Developing Advanced Technologies, Tehran, Iran; 4 Department of Material Science and Engineering, Faculty of Engineering, Arak University, Arak, 38156-8-8349 Iran; 5 International Center for Personalized Medicine, Düsseldorf, Germany; 6 Colorectal Research Center, Iran University of Medical Sciences, Tehran, Iran; 7 Breast Disease Research Center, Cancer Institute, Tehran University of Medical Sciences, P.O.: 1419733141, Tehran, Iran

**Keywords:** Cancer, Cell biology, Molecular biology, Oncology, Nanoscience and technology

## Abstract

Successful cancer treatment using magnetic hyperthermia therapy (MHT) strongly depends on biocompatible magnetic nanoparticles (NPs). They can effectively accumulate in tumor tissues after systemic injection and generate heat in the therapeutic temperature range (42–48 °C) by exposure to an AC magnetic field (AMF). For this purpose, folic acid-conjugated dextran-coated Zn_0.6_Mn_0.4_Fe_2_O_4_ (FA-Dex-ZMF) NPs were synthesized as smart nano heaters with self-regulating temperatures for MHT of liver tumors. Animal studies on BALB/c mice showed that the prepared NPs did not cause acute toxicity upon administration up to 100 mg kg^−1^. Likewise, no significant changes in hematological and biochemical factors were observed. FA-Dex-ZMF NPs were studied by exposing them to different safe AC magnetic fields (f = 150 kHz, H = 6, 8, and 10 kA m^−1^). Calorimetric experiments revealed that the NPs reached the desired temperature range (42–48 °C), which was suitable for MHT. Moreover, the efficacy of FA-Dex-ZMF NPs in MHT of liver tumors was investigated in vivo in liver-tumor-bearing mice. The obtained results revealed that the average volume of tumors in the control group increased 2.2 times during the study period. In contrast, the tumor volume remained almost constant during treatment in the MHT group. The results indicated that folic acid-conjugated dextran-coated Zn_0.6_Mn_0.4_Fe_2_O_4_ NPs with self-regulating temperature could be a promising tool for systemically delivered MHT.

## Introduction

Nowadays, magnetic nanoparticles (MNPs) have attracted much attention due to their potential applications in various fields of pharmacology and medicine, including drug delivery systems^1–3^, immunoassay^4^, magnetic hyperthermia therapy (MHT)^5,6^, and magnetic resonance imaging^7,8^. MHT, as a novel cancer treatment method, has been studied in vivo to treat various cancers, including lung, breast, prostate, head and neck, brain, pancreas, and liver^9–15^. The scientific basis behind this treatment method is a meager survival rate for cancer cells above 42 °C. The required heat in this process is supplied by magnetic nanoparticles, which transform magnetic energy into heat by exposing it to a noninvasive AC magnetic field (AMF). Moreover, it has also been found that MHT at mild temperatures (40–42 °C) can increase cancer cells' susceptibility to other treatments, such as chemotherapy and radiotherapy^16,17^. In Europe, MHT was approved as adjuvant therapy for recurrent glioblastoma multiform in combination with radiotherapy^18^. The application of MHT for cancer treatment is currently limited to accessible and localized tumors that can receive adequate nanoparticles by direct injection^19,20^. On the other hand, to prepare a sufficient concentration of MNPs in tumor tissue by intravenous injection, an extremely high dose of commotional Fe_3_O_4_ nanoparticles must be injected (1700 mg Fe/kg)^19^. This issue can be due to the non-targetability and relatively low tumor accumulation of conventional Fe_3_O_4_ nanoparticles following systemic injection. Therefore, designing MNPs with systemically delivered ability is essential for MHT applicability in the treatment of various types of tumors of different shapes and sizes. Moreover, nanoparticle systemic administration [intravenous (IV) or intraperitoneal (IP)] is minimally invasive compared to direct injection.

One of the most effective strategies to improve nanoparticle accumulation in tumor tissues is conjugating cancer cells by recognizing ligands on the surface of nanoparticles. This leads to nanoparticle uptake by tumor tissues. One of the most promising candidates for targeting nanoparticles to folate receptor overexpressing cancer cells is the conjugation of folic acid (FA) on the surface of nanoparticles^21^. Another approach to increasing the concentration of MNPs in tumor tissue is consecutive systemic injections at a safe dose. This can create an appropriate concentration of MNPs in tumor tissue^22,23^. For instance, Xie et al. reported that arginine-glycine-aspartic acid-targeted Mn-Zn ferrite magnetic nanocrystals could increase tumors' temperature to ~ 40 °C following a single intravenous injection of nanoparticles^23^. On the other hand, six repeated nanoparticle injections are required to improve the average tumor temperature to approximately 43–44 °C and significantly inhibit tumor growth^23^.

The most common magnetic nanoparticles introduced in biomedical applications are magnetite (Fe_3_O_4_) or maghemite (γ-Fe_2_O_3_) with high intrinsic Curie temperature (Tc) (T_C, Fe3O4_ = 585.1 °C and T_C,γ-Fe2O3_ = 447.1 °C). By exposing these nanoparticles even to a safe alternating magnetic field, it is impossible to adjust the maximum temperature created by nanoparticles to the desired range. This leads to overheating of the surrounding tissues during MHT. This problem can be solved by designing substituted ferrite nanoparticles with low Curie temperatures in the desired temperature range. When a magnetic nanoparticle is exposed to an AC magnetic field, it can generate heat if its temperature is lower than Tc. When the temperature reaches Tc, the heat generation mechanism stops. Therefore, Tc can act as an automatic switch for controlling magnetic nanoparticle maximum temperature. Until now, various types of magnetic nanoparticles with low Curie temperature were introduced for self-regulating MHT, including La–Sr–MnO_3_ perovskite oxide nanoparticles^5,6^, Co–Zn ferrite nanoparticles^24^, Mn–Zn ferrite nanoparticles^25^, Cu–Ni nanoparticles^26^, and Zn_0.54_Co_0.46_Cr_0.6_Fe_1.4_O_4_ nanoparticles^27,28^. Mn–Zn ferrite has attracted much interest because of the adjustable Curie temperature in the therapeutic temperature range by optimizing Mn or Zn doping, and the high specific absorption rate (SAR) compared to the other ferrites^23,29–31^. Moreover, Mn-Zn ferrite^'^s constituent elements (Fe, Mn, and Zn) are biocompatible. In recent years, numerous studies have investigated the heating ability of Mn_1-x_Zn_x_Fe_2_O_4_ nanoparticles (NPs) for MHT applications^30–32^. For example, Zn_0.6_Mn_0.4_Fe_2_O_4_ nanoparticles with the desired Curie temperature could be used in MHT. Their use led to cancer cells dying up to 90% within 15 min^29^.

This study synthesized dextran-coated Zn_0.6_Mn_0.4_Fe_2_O_4_ NPs (Dex-ZMF NPs) using the co-precipitation method followed by the hydrothermal process at 180 °C to produce biocompatible and colloidal stable Dex-ZMF NPs with heat generation capability under a safe AMF. Then, FA was conjugated on the surface of Dex-ZMF NPs to improve their targetability to cancer cells with folate receptors. The acute toxicity of the folic acid-decorated Dex-MZF NPs (FA-Dex-ZMF NPs) was evaluated by administering different doses of nanoparticles to BALB/c mice, and hematological/biochemical parameters were monitored. Besides, the therapeutic efficacy of FA-Dex-ZMF NPs for MHT of liver tumors was investigated in an animal tumor model of liver cancer. To the best of our knowledge, this is the first study of systemically delivered magnetic nanoparticles for magnetic hyperthermia therapy of liver tumors.

## Results and discussion

**Figure 1:**
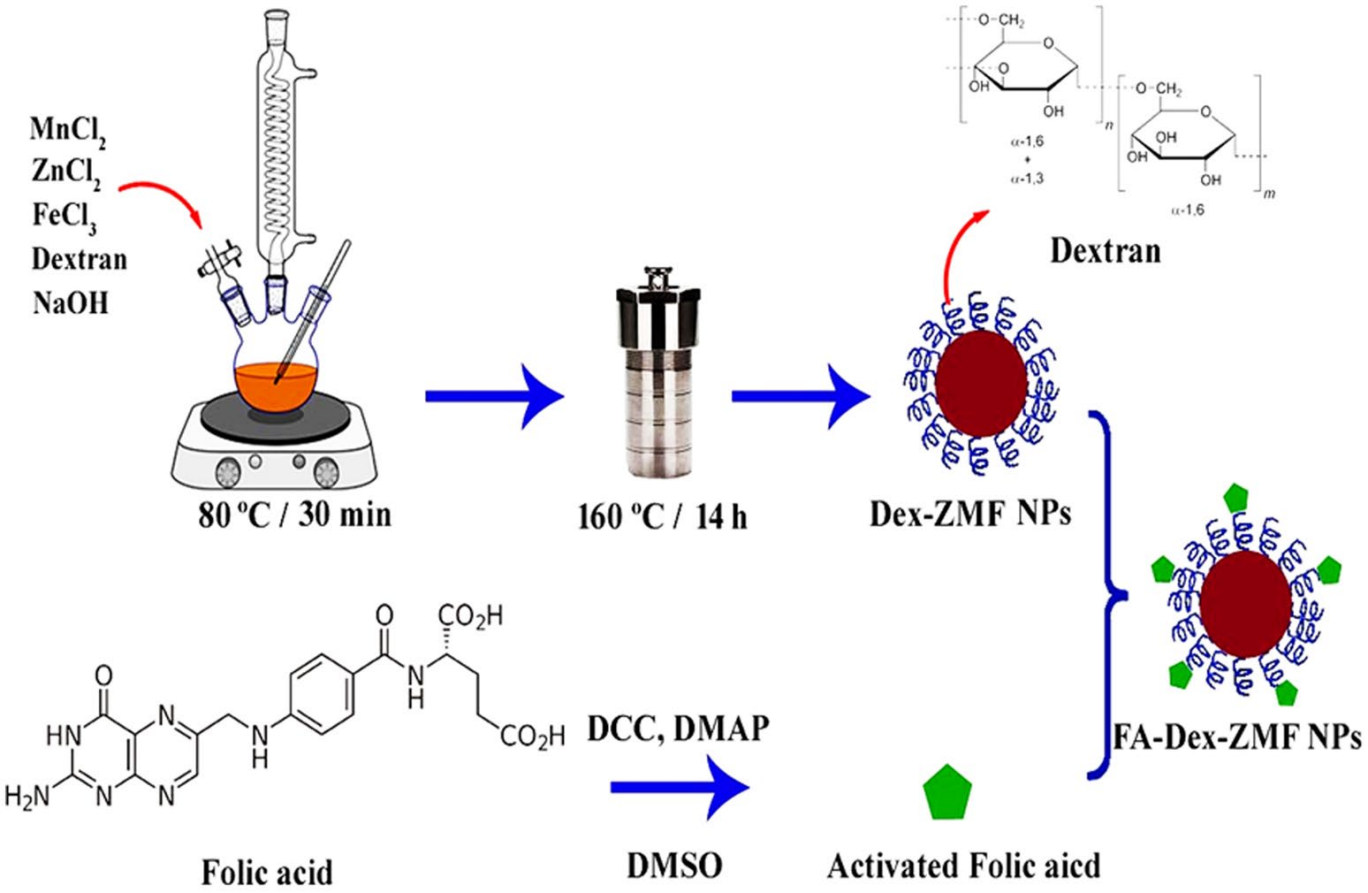
Synthesis process for FA-Dex-ZMF NPs.

Different chemical methods have been introduced for synthesizing magnetic nanoparticles^33–35^. Surface modification of magnetic nanoparticles is vital to enhance their biocompatibility and colloidal stability in physiological media. In this study, dextran-coated Zn_0.6_Mn_0.4_Fe_2_O_4_ nanoparticles were synthesized by the co-perception method followed by the hydrothermal process to promote nanoparticle magnetic properties^22^. Finally, to enhance nanoparticle tumor-targeting ability, FA was conjugated to Dex-ZMF NPs. Figure 1 shows the synthesis process of FA-Dex-ZMF NPs schematically.

**Figure 2:**
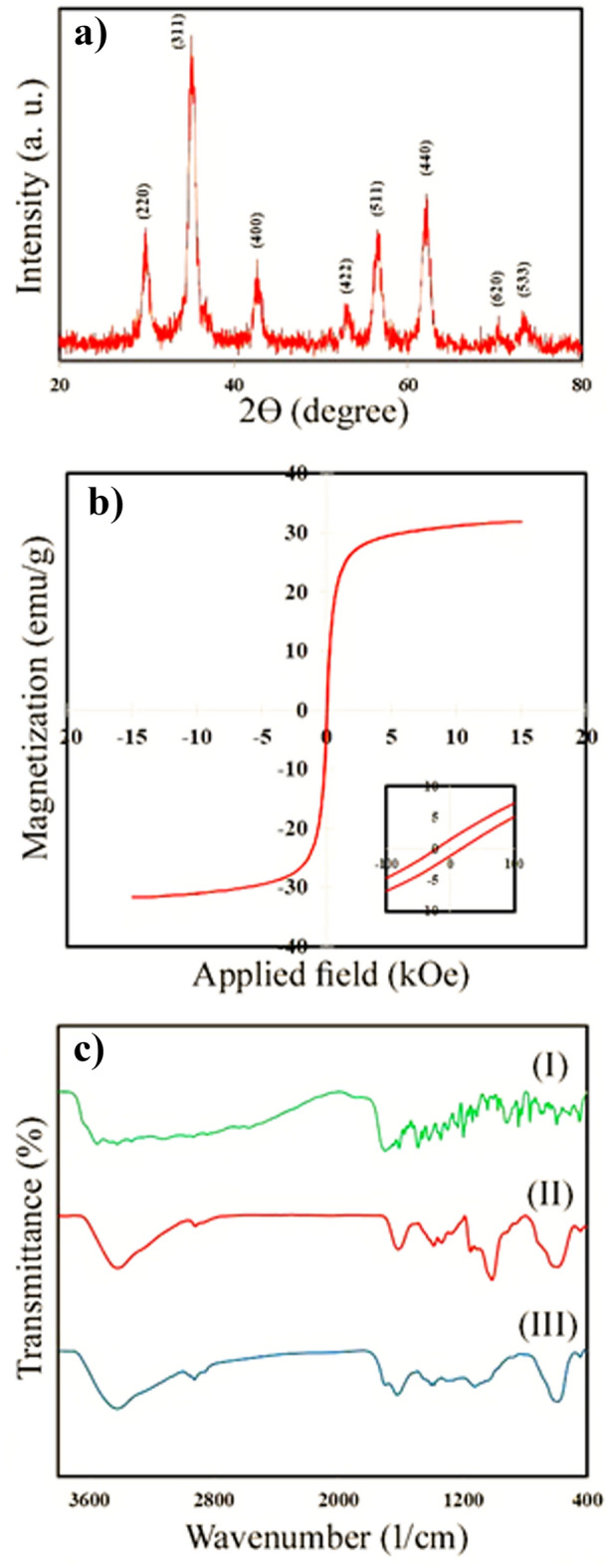
(**a**) XRD pattern of Dex-ZMF NPs, (**b**) magnetization curve of FA-Dex-ZMF NPs, the inset exhibits the hysteresis loop at low fields, and (**c**) FTIR spectra of (I) FA, (II) Dex-ZMF, and (III) FA-Dex-ZMF NPs.

XRD analysis was used to investigate the crystalline structure of Dex-ZMF NPs after hydrothermal treatment, and the obtained XRD pattern is shown in Fig. 2a. Characteristic peaks observed at the planes (220), (311), (400), (511), and (440), correspond well with JCPDS File No. 74-2401, indicating the formation of a single cubic spinel structure for Zn_0.6_Mn_0.4_Fe_2_O_4_ NPs. The average crystallite size (D) of Zn_0.6_Mn_0.4_Fe_2_O_4_ NPs was calculated using Debye's Scherrer formula:

1 $$D=0.9\lambda /\beta cos\theta$$

where λ represents the wavelength of the incident X-ray beam, θ represents the diffraction angle of the most intense peak, and β is the full width at half maximum (FWHM). Experimental data showed that the mean crystallite size of Zn_0.6_Mn_0.4_Fe_2_O_4_ NPs was 13.5 nm.

The saturation magnetization and hysteresis loops of FA-Dex-ZMF NPs were measured at 300 K using VSM analysis (Fig. 2b). The sample's saturation magnetization (Ms) and coercivity (Hc) were 31.8 emu/g and 18.5 Oe, respectively. The inset of Fig. 2b shows an enlarged view of the hysteresis loop in which the sample's Hc and remanent magnetization (Mr) can be observed. It has been found that magnetic nanoparticles in the superparamagnetic regime have zero coercivity^36^. By increasing the nanoparticle size, it can leave the superparamagnetic regime and enter the ferromagnetic regime. Therefore, Hc appears in the hysteresis loop. According to this study, the prepared nanoparticles are ferromagnetic.

FTIR analysis confirmed the spinel structure of ZMF NPs. It also coated dextran on the ZMF NP surface and further altered the nanoparticle's surface with FA. The FTIR spectra of Dex-ZMF NPs, FA-Dex-ZMF NPs, and pure FA are shown in Fig. 2c. As can be observed, several characteristic bands can be seen in FA's FTIR pattern. The bands that appear at 3560 and 3431 cm^-1^ can be attributed to the stretching vibration of hydroxyl groups (–OH) and N–H bands, respectively. Also, CH_2_ stretching vibrations at 2930 and 2851 cm^−1^ can be observed. In addition, the strong absorption band at 1705 cm^−1^ can be related to the stretching vibration of C=O in the glutamate moiety. The band that appears at 1607 cm^−1^ can be assigned to the bending vibration of the –NH bond^27^.

In the FTIR spectra of Dex-ZMF NPs, the two appearing bands in the range of 400–900 cm^−1^ are due to stretching vibrations of metal–oxygen bonds^37^. Moreover, the absorption bands at 2931 and 1353 cm^−1^ can be assigned to the stretching and bending vibration of –CH_2_– in the dextran coating. In addition, the bands exhibited at 1153 and 1023 cm^−1^ can be assigned to the stretching vibration of hydroxyl groups (–OH) in the dextran structure. These results confirmed that ZMF NPs were successfully coated with dextran. In the FTIR pattern of FA-Dex-ZMF NPs all characteristic bands of FA and Dex-ZMF NPs are present. After conjugation, the characterized peak of FA's carbonyl group (C=O) became weaker. It underwent a minor redshift to 1690 cm^−1^, confirming the conjugation between FA and dextran's hydroxyl group.

**Figure 3:**
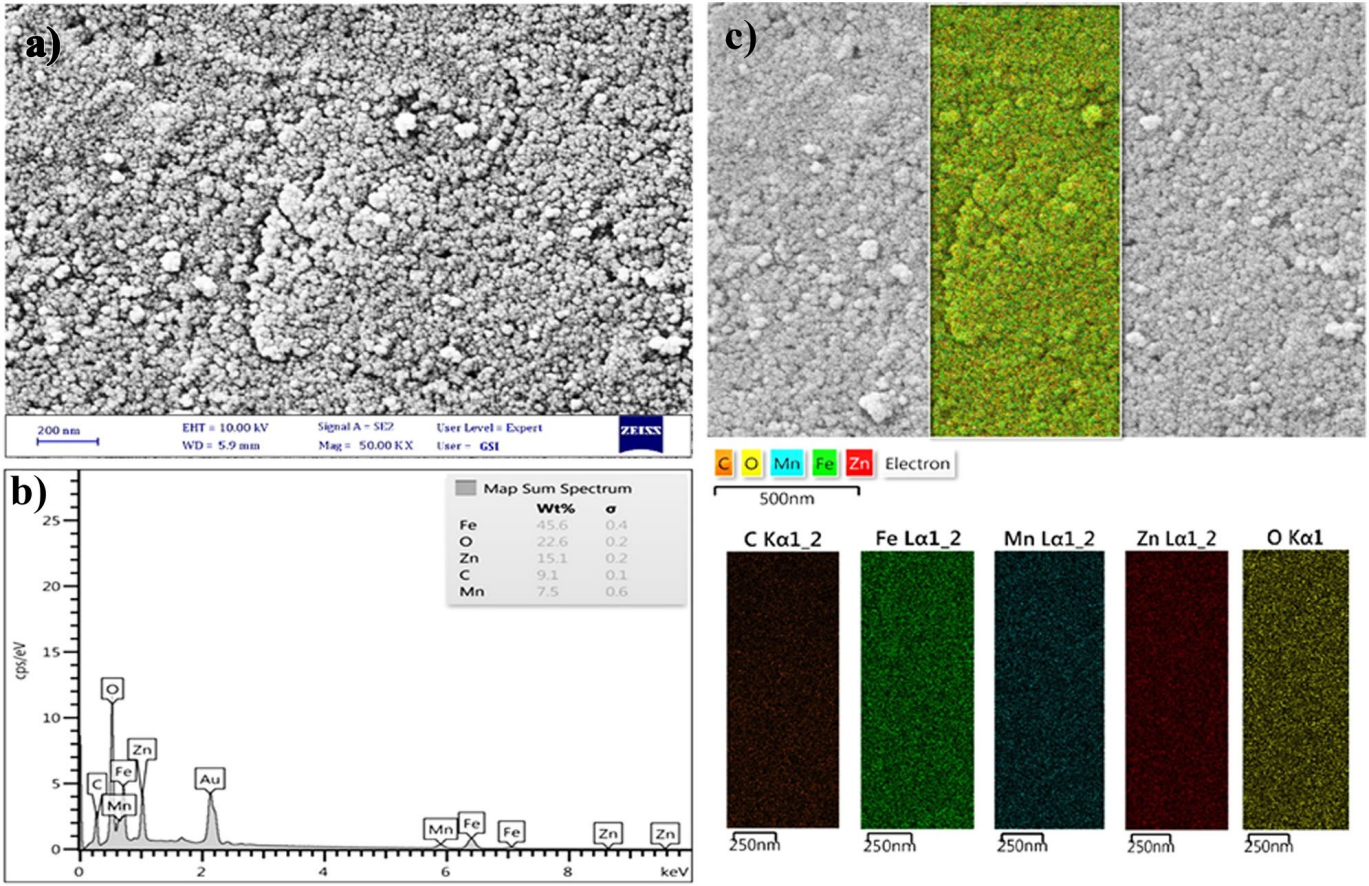
(**a**) SEM image of FA-Dex-ZMF NPs, (**b,c**) EDX and mapping analyses of FA-Dex-ZMF NPs.

FA-Dex-ZMF NPs were investigated by FESEM analysis (Fig. 3a). The prepared sample showed an almost spherical shape in the nanoscale regime. The mean sample particle size was 15–30 nm. It has been reported that nanoparticles in the range of 10–100 nm have the longest blood circulation time. FA-Dex-ZMF NPs prepared in our study had suitable dimensions for biomedical applications^38^.

Energy-dispersive X-ray spectroscopy (EDX) analysis (Fig. 3b) showed the presence of the major elements in the desired ratio in Zn_0.6_Mn_0.4_Fe_2_O_4_ nanoparticles. Likewise, the elemental mapping analysis (Fig. 3c) exhibited a uniformly homogenous distribution for Fe, Mn, and Zn elements in the sample, suggesting uniformity of products. Moreover, the presence of the C element in a sample was attributed to the presence of carbon elements in the dextran and folic acid molecules on the surface of nanoparticles.

Nanoparticle surface charge plays a critical role in colloidal stability and interaction with physiological cells^39,40^. The prepared Dex-ZMF NPs showed a negative surface charge (−17.1 mV) which may be due to the presence of terminal hydroxyl groups in the dextran coating structure. Moreover, after surface modification of NPs by FA, a more negative surface charge (−22.5 mV) was observed, which may be due to the addition of a negative charge related to the carboxyl group of FA molecules conjugated on the surface of NPs^41^. These results showed that the prepared nanoparticles are physically stable and can escape from the reticuloendothelial system (RES) and reach cancer cells through the EPR effect^39^.

To evaluate the heating efficacy of FA-Dex-ZMF NPs in magnetic fluid hyperthermia, the solution containing FA-Dex-ZMF NPs (5 mg/mL) was exposed to an AMF with different amplitudes (H = 6, 8, and 10 kA m^−1^). The frequency of all magnet fields was fixed at 150 kHz, and the time-dependent temperature curve of the solution in each experiment was recorded. The amplitude and frequency of the applied AMF were selected to meet the safety limit for magnetic hyperthermia applications (H × f < 5 × 10^9^ A m^−1^ s^−1^)^36,42^. In the in vitro analyses of magnetic hyperthermia, the magnetic fluid concentration was selected to be less than 10 mg/mL due to negligible cytotoxicity in cell culture^29,43^. On the other hand, it has been shown that the optimum concentration of magnet nanoparticles in a ferrofluid to achieve maximum SAR is 1–10 mg/mL^44^. Thus, in our study, 5 mg/mL concentration was chosen for in vitro and in vivo hyperthermia experiments.

**Figure 4:**
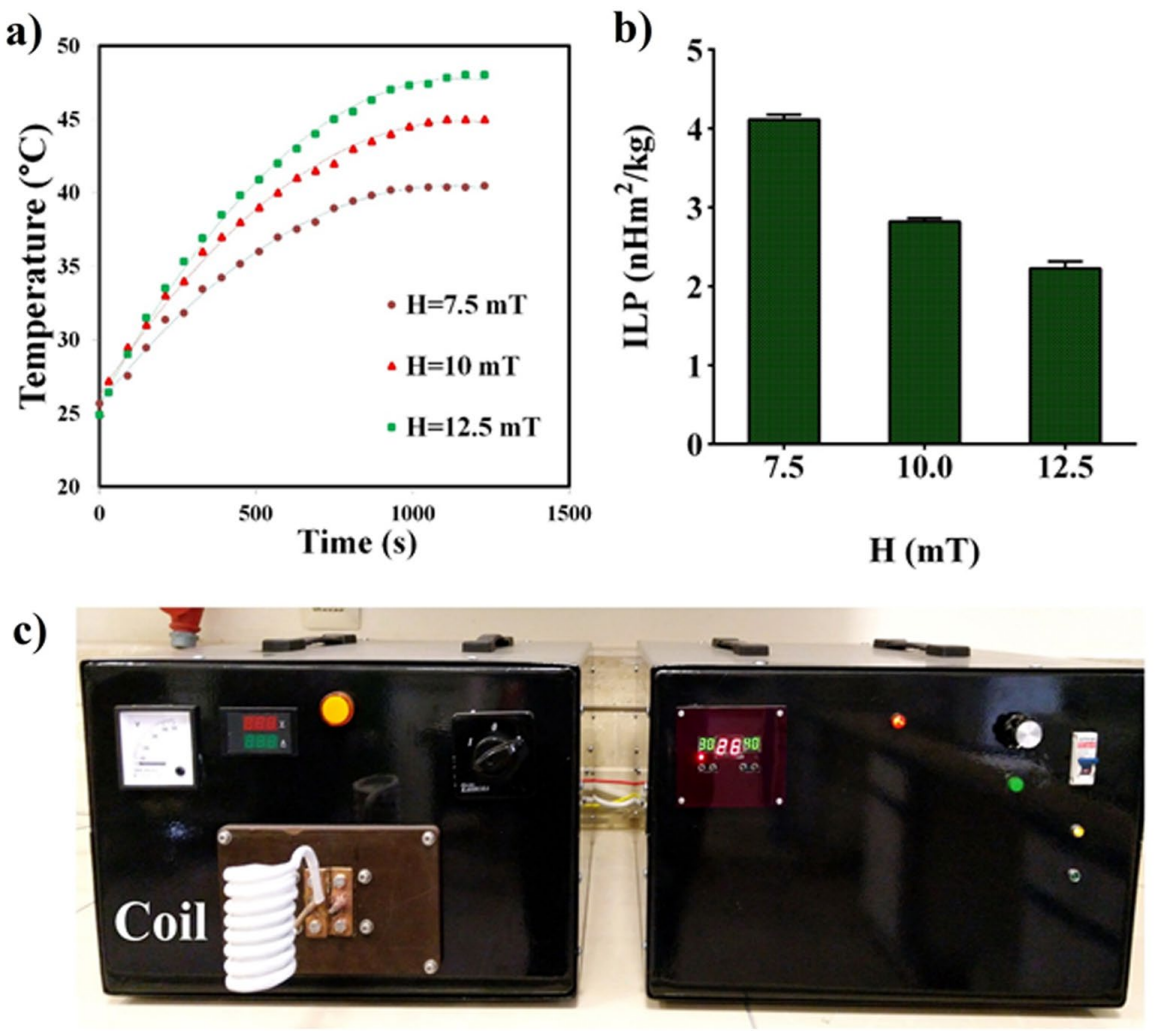
(**a**) Time-dependent temperature curves for Dex-MZF NPs at different magnetic field amplitudes, (**b**) ILP values calculated at different magnetic field amplitudes, (**c**) instrument used for in vitro and in vivo MHT experiments.

Figure 4a shows the time-dependent temperature curves of FA-Dex-ZMF NPs in solution, and the values of calculated intrinsic loss power (ILP) according to Eq. (2) are displayed in Fig. 4b. By enhancing the magnetic field amplitude from 7.5 to 12.5 mT, the ILP values decrease from 4.1 to 2.4 nHm^2^/kg (Fig. 4b). Moreover, the mean ILP values of Dex-ZMF NPs are about 25 and 50 times larger than those reported for Feridex (0.15 nHm^2^/kg) and Resovist (0.07 nHm^2^/kg) nanoparticles (two commercial Fe_3_O_4_ nanoparticles), respectively^45,46^.

Moreover, the maximum temperature attained by FA-Dex-ZMF NPs in powder form, which is a rough estimate of the Curie temperature, was determined by exposing the dry powder of FA-Dex-ZMF NPs to an AMF (150 kHz; 12.5 mT). The temperature of the FA-Dex-ZMF NPs in the powder form rapidly increased under the AC magnetic field (Fig. 4c), and it became almost saturated after 10 min around 73 °C, very close to the Curie temperature of the Mn_0.4_Zn_0.6_Fe_2_O_4_ nanoparticles reported by other researchers^25,31^.

### In vivo toxicity and targetability of FA-Dex-ZMF NPs

**Figure 5:**
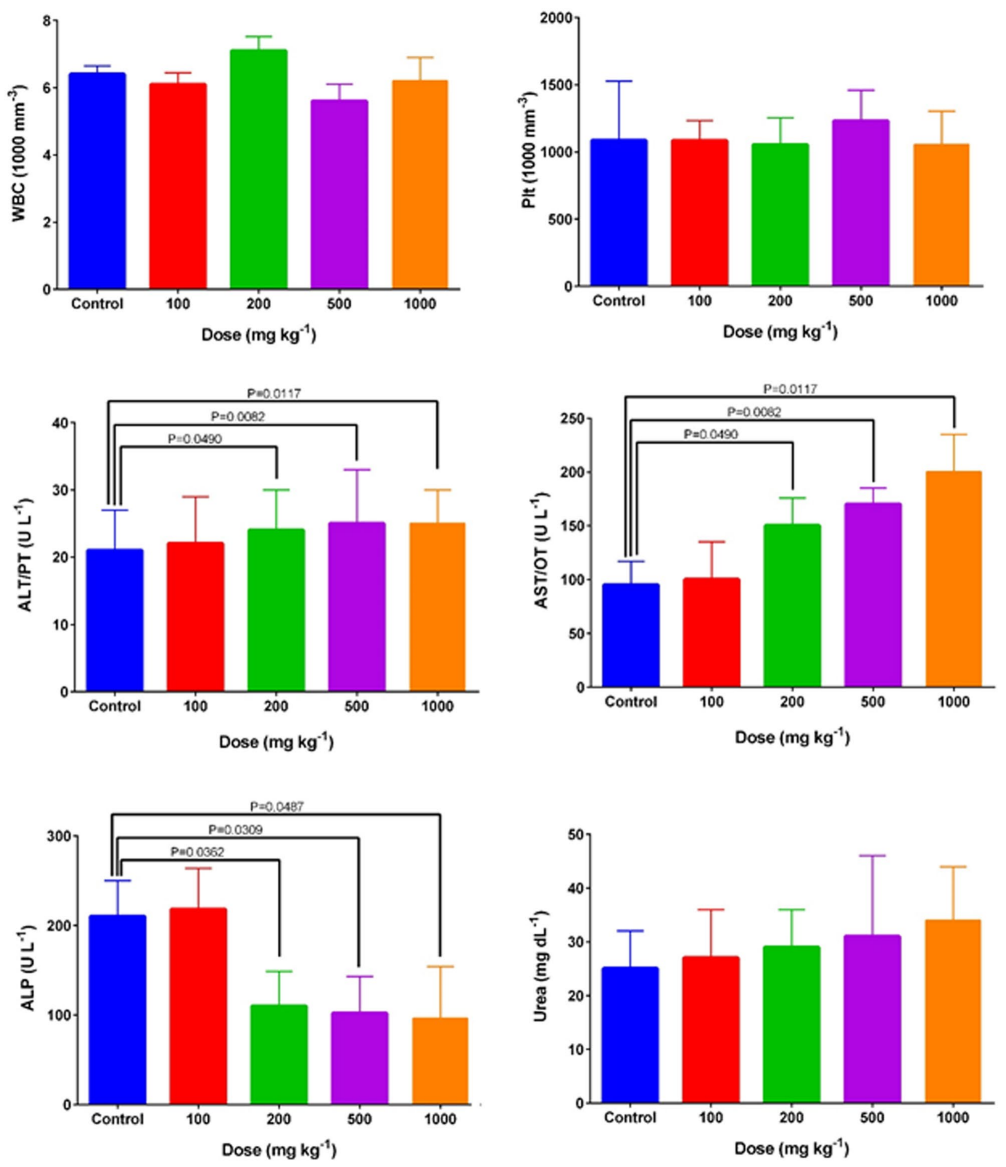
Major hematological and blood chemical indices in BALB/c mice after a single dose of FA-Dex-ZMF NPs. *WBC* white blood cells, *Plt* platelets, *urea* blood urea nitrogen, *AST* aspartate transaminase, *ALT* alanine transaminase, and *ALP* alkaline phosphatase.

The main results of the acute toxicity evaluations of FA-Dex-ZMF NPs on hematological/clinical parameters are shown in Fig. 5, and details are reported in Table 1.

**Table 1:** Acute toxicity effects of FA-Dex-ZMF NPs on hematological and biochemical indices.

Parameters	Control	100 mg kg^−1^	200 mg kg^−1^	500 mg kg^−1^	1000 mg kg^−1^
White blood cells (WBC)	6.4 ± 0.25	6.1 ± 0.35	7.1 ± 0.32	5.6 ± 0.50	6.2 ± 0.70
% Lymph	63 ± 16	62 ± 20	65 ± 16	70.5 ± 9	71 ± 10
Red blood cells (RBC)	7.2 ± 1.5	7.1 ± 2.5	6.9 ± 2.2	7.1 ± 1.2	6.8 ± 1.6
Hemoglobin (Hgb)	11.8 ± 2	11.6 ± 1.5	11.5 ± 1.2	11.8 ± 1.3	10.1 ± 2.3
Hematocrit (HCT)	36 ± 5.7	34 ± 6	34.5 ± 5	35.6 ± 3.5	31.9 ± 7
Mean corpuscular volume (MCV)	49.8 ± 5.9	51 ± 7.0	50 ± 6.0	51 ± 5.0	50 ± 4.0
Mean corpuscular hemoglobin (MCH)	15.6 ± 2.5	15.5 ± 3	15.8 ± 2	16 ± 2.3	15.9 ± 3
Platelets (PLT)	1070 ± 465	1083 ± 150	1053 ± 201	1230 ± 230	1054 ± 250
Platelet distribution width (PDW)	8.2 ± 2.6	8.2 ± 2	8 ± 1	8.2 ± 1.4	8.5 ± 2
Mean platelet volume (MPV)	6.8 ± 1.9	6.7 ± 0.8	6.9 ± 0.7	7 ± 0.8	7 ± 0.9
Blood urea nitrogen (BUN)	25 ± 7	27 ± 9	29 ± 7	31 ± 15	34 ± 10
Alkaline phosphatase (ALP)	210 ± 40	218 ± 46	110 ± 39*	102 ± 41*	96 ± 58*
Aspartate transaminase (AST/OT)	95 ± 22	100 ± 35	150 ± 26*	170 ± 15*	200 ± 35*
Alanine transaminase (ALT/PT)	21 ± 6	22 ± 7	24 ± 6*	25 ± 8*	25 ± 5*
Glucose (GLU)	78 ± 12	85 ± 35	80 ± 40	85 ± 20	85 ± 19
Calcium (Ca)	3.6 ± 1	3.5 ± 0.7	3.7 ± 0.4	3.6 ± 0.7	3.8 ± 0.8
Magnesium (Mg)	0.95 ± 0.1	1.1 ± 0.6	1.2 ± 0.5	1.2 ± 0.6	1.3 ± 0.7
Direct Bilirubin (D.Bil)	0.04 ± 0.03	0.05 ± 0.01	0.05 ± 0.01	0.07 ± 0.04	0.08 ± 0.03
Total protein (TP)	1.4 ± 0.1	1.2 ± 0.5	1.3 ± 0.3	1.2 ± 0.2	1.4 ± 0.4

Values are reported as means ± SD, and (*) indicates a significant change in parameters compared to the control group (P < 0.05).

Liver enzyme activities such as ALT, AST, and ALP are significant characteristics of liver function. Blood urea nitrogen is the principal characteristic of kidney function. No Dex-ZMF NPs-associated death or severe poisoning symptoms were observed in any of the acute toxicity phases. As seen in Fig. 5, the animals acutely treated with Dex-ZMF NPs at all doses greater than 100 mg kg^−1^ demonstrated a significant change in the main liver enzymes compared to the “Control group” (P < 0.05). ALP decreased; on the other hand, ALP and AST increased with increasing NPs doses (Fig. 5).

Before MHT experiments, it is essential to ensure that the prepared NPs can accumulate in the tumor tissues. This will provide adequate concentrations for effective heat generation at the target site. To this end, the concentration of Fe ion as a major component in the prepared NPs was measured in the tumor tissues of the “control group” and “NPs group” (mice in the “NPs group” received three injections of NPs (50 mg kg^−1^) with a 24 h interval) using ICP-MS analysis. The results revealed that the average concentration of Fe in the tumor tissues of the Control group is 0.7 mg_Fe_/g_Tumor_, compared to 1.88 mg_Fe_/g_Tumor_ for the NPs group (P < 0.05). According to the ICP-MS analysis results, after three systemic injections of FA-Dex-ZMF NPs, about 16 wt% of the total amount of injected NPs accumulated in the tumor tissue.

### In vivo MHT experiments

**Figure 6:**
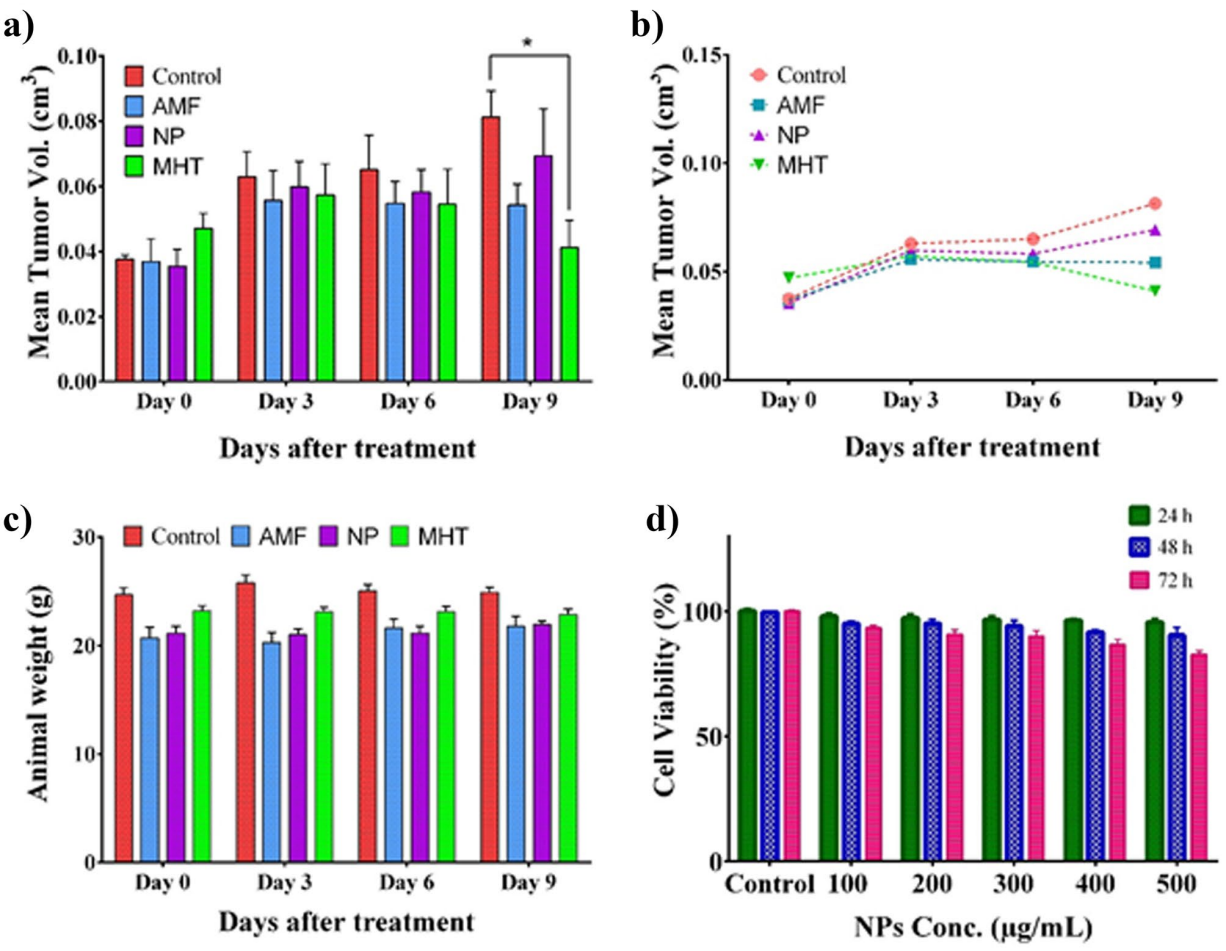
Time courses of tumor growth during treatment as (**a**) bar graph, and (**b**) line graph. (**c**) Body weight changes of the mice without treatment (control group), treated by intravenous injection of FA-Dex-ZMF NPs (NP group), exposed to an AC magnetic field (AMF group), and treated group with a combination of intravenous injection of FA-Dex-ZMF NPs and AC magnetic field (MHT group), and (**d**) the cytotoxicity of FA-Dex-ZMF NPs at different concentrations and times after incubation with MC4L2 cells, (*) indicates P-value < 0.05.

FA-Dex-ZMF NPs were evaluated in liver tumor-bearing mice. No groups observed mortality or significant behavior changes during the experiments. Figure 6a shows the average tumor volume in each group during treatment. A considerable difference between the tumor volume in the “MHT group” and the “control group" can be observed. As seen in the control group, the average tumor volume increased from 0.038 to 0.081 cm^3^ during nine days of treatment. In contrast, the average tumor volume decreased from 0.047 to 0.041 cm^3^ in the MHT group. This indicates MHT's effectiveness at controlling tumor size (Fig. 6a). In other words, the average volume of tumors in the “control group” increased up to 2.2 times during the study period. In contrast, the tumor volume remained almost constant in the “MHT group”. In summery, a significant decrease in the tumor volume can be observed in the “MHT group” compared to the “Control group” on the 9th day of treatment (P < 0.05). Moreover, the mean final volume of tumors in the NPs and AMF groups was higher than in "The MHT group". This indicates FA-Dex-ZMF NPs and AMF alone could not suppress tumor growth (Fig. 6b). Using the nanoparticles synthesized in this study, we demonstrated MHT's effectiveness in controlling hepatocellular carcinoma.

One of the critical factors for identifying and ensuring the biocompatibility of nanoparticles or applied magnetic fields in the internal environment is monitoring animals' weight, appearance, and grooming. If nanoparticles or external magnetic fields negatively affect mice, their weight will be significantly reduced. Here, we evaluated the impact of external stimuli and the method of treating mice' weight in all groups (Fig. 6c). As can be observed, the weight of mice in all groups did not decrease. This indicates the non-toxicity of nanoparticles at the recipient dose and the lack of destructive effects of the magnetic field applied to mice. For the clinical application of MHT, there are two strict and less strict criteria for the product of the intensity (H) and frequency (f) of the applied magnetic field which is called the Atkinson–Brezovich limit (H × f = 4.85 × 10^8^ A m^−1^ s^−1^), and the Hergt’s limit (H × f = 5 × 10^9^ A m^−1^ s^−1^), respectively. In our experiment, a safe magnetic field (H = 10 kA m^−1^, and f = 150 kHz) was used in all experiments (H × f = 1.5 × 10^9^ kA m^−1^ was less than Hergt’s limit)^36,42^. Moreover, the cytotoxicity of FA-Dex-ZMF NPs against MC4L2 cells was measured by MTT assay (Fig. 6d). FA-Dex-ZMF NPs with different concentrations of 100, 200, 300, 400, and 500 µg mL^−1^ were exposed to MC4L2 cells for 24, 48, and 72 h. As shown in Fig. 6d, although the survival of MC4L2 cells was decreased with increasing the concentration of FA-Dex-ZMF NPs in the medium, the concentration of 500 μg mL^−1^ of the prepared nanoparticles is still safe.

## Conclusions

Our study showed that FA-Dex-ZMF NPs could produce adequate heat at the desired temperature range upon exposure to an AC magnetic field without overheating. We observed no acute toxicity in mice upon nanoparticle administration up to 100 mg kg^−1^. No significant changes in hematological and biochemical factors were observed. Likewise, MHT using FA-Dex-ZMF NPs on mice with hepatocellular carcinoma tumors showed this treatment's capability to control tumor volume. It has been found that repeated MHT using designed nanoparticles suppresses tumor growth. The essential advantage of the proposed nanoparticles in this study was the ability to be injected into the body without apparent toxicity in mice. Further experiments and analyses are planned to evaluate the therapeutic outcomes of the proposed method in combination with other anti-cancer therapies.

## Materials and methods

### Materials

All chemicals were used without further purification and were analytical grade. Anhydrous dimethyl sulfoxide (DMSO), iron chloride tetrahydrate (FeCl_2_⋅4H_2_O), iron chloride hexahydrate (FeCl_3_⋅6H_2_O), manganese chloride tetrahydrate (MnCl_2_⋅4H_2_O), zinc chloride tetrahydrate (ZnCl_2_⋅4H_2_O), dextran (Mw ≈ 10,000), folic acid (FA), *N, N'*-dicyclohexylcarbodiimide (DCC), 4-dimethylamino pyridine (DMAP), and sodium hydroxide (NaOH) were purchased from the Sigma-Aldrich Company. Deionized water was used throughout the experiments. The Heap1-6 cells were prepared at the Pasteur Institute of Iran (Tehran, Iran).

### Synthesis of Dex-ZMF NPs

Dextran-coated Zn_0.6_Mn_0.4_Fe_2_O_4_ NPs were synthesized by the co-perception method followed by hydrothermal processes^47^. To this end, metal cations in a stoichiometric ratio according to the desired final product composition were mixed in a dextran solution. This was done in nitrogen. The mixture's temperature was increased to 80 °C. Sodium hydroxide solution (1 M) was rapidly added, stirring the mixture for 30 min. The change in solution color from light brown to black indicated Zn_0.6_Mn_0.4_Fe_2_O_4_ nanoparticle formation. Then, the black suspension was poured into a sealed autoclave and heat treated at 180 ºC for 12 h. The obtained product was then washed using the ultra-centrifugation method.

### Synthesis of FA-Dex-ZMF NPs

The surface modification of Dex-ZMF NPs with folic acid was conducted by conjugating the carboxyl group of the FA to the hydroxyl group of dextran via esterification reaction^48^. To activate the carboxyl group of folic acid, 0.005 g of DMAP, 0.01 g of DCC, and 0.02 g of folic acid were dissolved in DMSO. The mixture was stirred at room temperature for 24 h in an N_2_ atmosphere. Then, Dex-ZMF NPs (5 mg ml^−1^) were added to the reaction mixture, and stirring was continued for another 24 h at 80 °C in darkness. The obtained product was washed with water and ethanol using ultra-centrifugation.

### Characterization of FA-Dex-ZMF NPs

The crystalline structure of FA-Dex-ZMF NPs was studied by powder X-ray diffraction (XRD, Philips, X-pert) analysis with Cu-K radiation through a Ni filter (λ = 0.15418 nm). Functional groups and conjugation of FA to nanoparticles were investigated using Fourier transform infrared spectroscopy analysis (FTIR, Bruker Vertex 70 spectrometer). FA-Dex-ZMF NPs were analyzed by field emission scanning electron microscopy (FESEM, ZEISS Sigma 300, Germany). The magnetic properties of FA-Dex-ZMF NPs were measured using a vibrating sample magnetometer (VSM) conducted at room temperature. The surface charge of the prepared nanoparticles was measured by Zeta potential analysis (DLS, Malvern, Zetasizer, UK).

### In vitro toxicity

The cytotoxicity of FA-Dex-ZMF NPs was measured on MC4L2 cells using the Methyl ThiazolTetrazolium Bromide (MTT) assay. To this end, approximately 1 × 10^4^ cells/well were cultured in a 96-well plate. Different concentrations of FA-Dex-ZMF NPs (100, 200, 300, 400, and 500 μg.mL^-1^) were added to each group of cells and the viability of cells was evaluated by the MTT method after 24, 48, and 72 h of post-treatment^18^.

### In vivo toxicity

Twenty-five BALB/c mice (6–8 weeks old, female) were randomly distributed into five groups (5 mice per each group) to assess the acute toxicity of FA-Dex-ZMF NPs. The first group did not receive any treatment and was considered the “Control group”. The second to fifth groups received FA-Dex-ZMF NPs intraperitoneally (IP) at 100, 200, 500, and 1000 mg kg^−1^, respectively. Animals were sacrificed under general anesthesia (xylazine 10 mg kg^−1^ and ketamine 100 mg kg^−1^) 24  h post-NPs injection, and blood samples were taken to measure hematology and clinical chemistry parameters. Animal body weight changes and abnormal hematological/biochemical indices were used as toxicity signs.

### ICP-MS analysis

To evaluate the targetability of FA-Dex-ZMF NPs to tumor tissue, the concentration of NP in the tumor tissue after injection was evaluated by inductively coupled plasma mass spectrometry analysis (ICP-MS). To this end, 12 breast tumor-bearing mice were randomly divided into two groups (six mice in each group) including (1) the “Control group” and (2) the “NPs group”. Mice in the “Control group” did not receive any treatment and served as the base concentration of Fe in the tumor tissue. Mice in the “NPs group” received three doses of the IP injection of FA-Dex-ZMF NPs (50 mg kg^−1^) at 24 h intervals. Mice were euthanized 24 h after the last injection, and their tumors were harvested. The Fe concentration as a major component in FA-Dex-ZMF NPs was measured in tumor tissues using ICP-MS^49^.

### Magnetic hyperthermia experiments

#### In vitro analysis

The heat generation ability of FA-Dex-ZMF NPs was investigated using a homemade induction heating unit equipped with a solenoid coil (8 turns, 4 cm diameter). In our experiments, an insulated microtube containing a 1.5 ml solution of FA-Dex-ZMF NPs (5 mg ml^−1^) was inserted in the center of the solenoid coil. By applying an AMF, the sample temperature was monitored versus time. The intrinsic loss power (ILP) of the sample was calculated according to equation^36^:

2 $$ILP\, \left(\mathrm{nH}\, {\mathrm{ m}}^{2} \, {\mathrm{kg}}^{-1}\right)=\frac{SAR}{f\times {H}^{2}}$$

where *f* represents the frequency and *H* is the amplitude of the AMF. Besides, SAR in Eq. (2) is the specific absorption rate of the sample, which can be determined as follows^6^:

3 $$SAR\, \left(\mathrm{W}\, {\mathrm{ g}}^{-1}\right)=\left({C}_{S}/{X}_{NP}\right)\left(dT/dt\right)$$

CS indicates the sample's specific heat capacity, and X_NP_ represents the weight fraction of ZMF NPs in the sample. Besides, ($dT/dt$) is the initial slope of the time-dependent temperature curve recorded in each experiment.

### In vivo analysis

To create liver tumor-bearing animals, on the right flank of 28 BALB/c mice, 100 µL PBS containing approximately 1 × 10^6^ Heap1-6 cells was injected subcutaneously under anesthesia. After 15 days, the liver tumors had grown to around 0.04 cm^3^, and tumor-bearing mice were enrolled in our experiments. Then, the mice were randomly distributed into four groups (7 mice in each group), including (1) the control group, (2) the NP group, (3) the AC magnetic field group (AMF group), and (4) the magnetic hyperthermia therapy group (MHT group). Figure 1 shows the treatment protocol for all the groups. It includes the number of times mice received FA-Dex-ZMF NPs and/or the number of times mice were exposed to the AC magnetic field.

**Figure 7:**
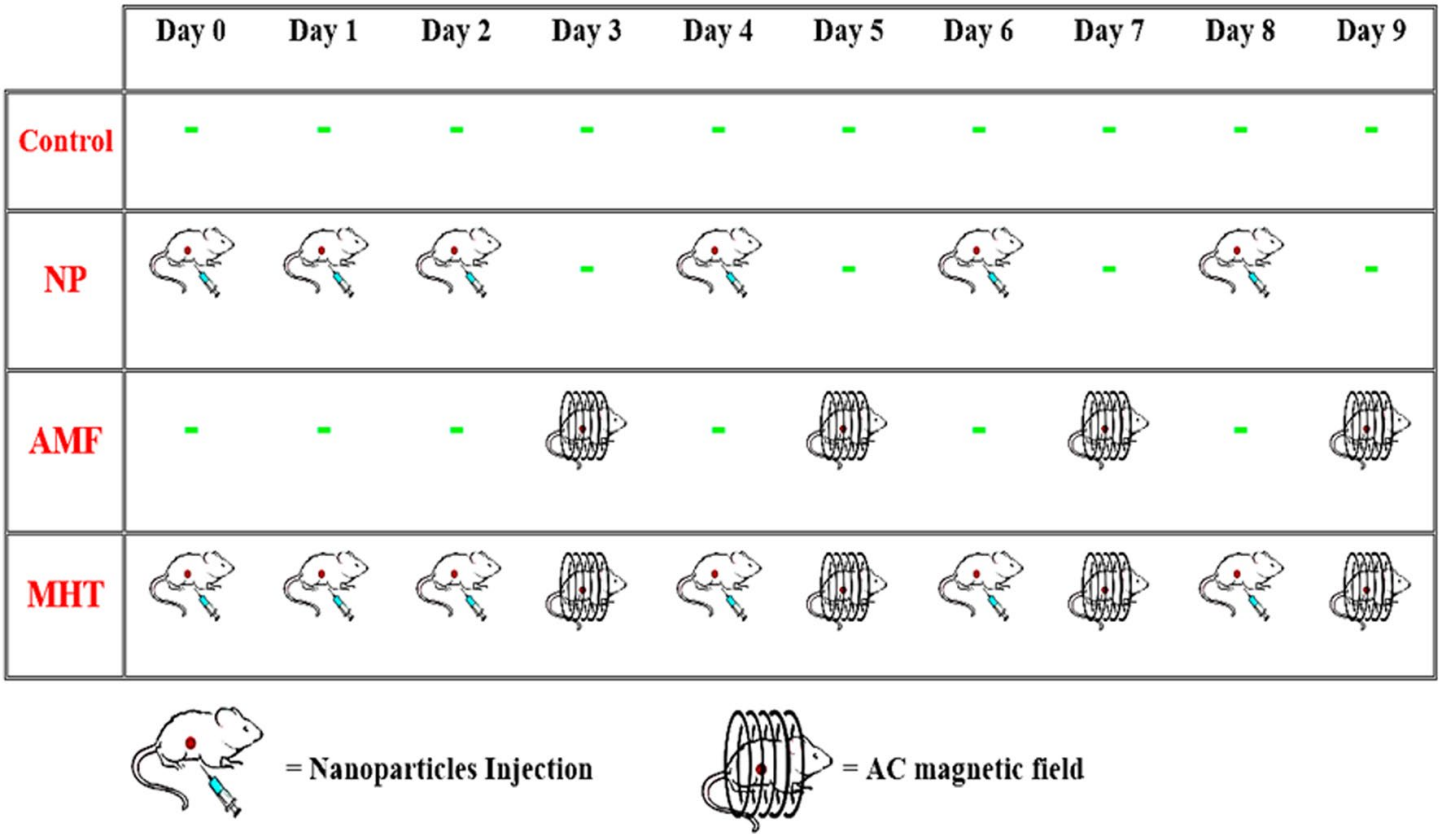
The treatment protocol for each study group.

As shown in Fig. 7, in the “Control group,” animals did not take any experimental treatment and were only considered a reference group to monitor normal tumor growth. Mice in the “NP group” received six consecutive doses of FA-Dex-ZMF NPs (each dose = 50 mg kg^−1^) on days 0, 1, 2, 4, 6, and 8 after tumor modeling. Mice in the “AMF group” were exposed to a constant AMF (ƒ = 150 kHz, H = 10 kA m^−1^) on days 3, 5, 7, and 9 after tumor modeling, without nanoparticle injection. Finally, in the “MHT group”, mice received six consecutive doses of FA-Dex-ZMF NPs (each dose = 50 mg kg^−1^) on days 0, 1, 2, 4, 6, 8, and were also exposed to AMF on days 3, 5, 7, and 9. Animals were anesthetized by IP injection of ketamine and xylazine before each experiment. The tumor volume was determined by the following formula:

4 $$\mathrm{V}=\frac{\uppi }{6}\times (\mathrm{LWD})$$

L, W, and D represent the tumor’s length, width, and depth.

### Statistical analysis

The experimental data were analyzed using GraphPad Prism software version 6.0. We used t-test and ANOVA to analyze parametric data in two and multi groups, respectively. A statistically significant change in data was considered when the P-value was less than 0.05.

### Ethical approval

All methods were performed under the relevant guidelines and regulations. All procedures performed in animal studies were conducted within the international guidelines of the Weatherall report and the national guidelines of the Institutional Animal Care and Use Committee (IACUC) of the Iran University of Medical Sciences (IUMS). The Iran University of Medical Sciences Ethics Committee has approved the project (No: IR.IUMS.RAHC.REC.25671). Reports concerning experimental animals follow the ARRIVE guidelines^50^.

## Data Availability

The data supporting this study's findings are available from the corresponding author, AMA, upon reasonable request.
